# ghr-miR5272a-mediated regulation of *GhMKK6* gene transcription contributes to the immune response in cotton

**DOI:** 10.1093/jxb/erx373

**Published:** 2017-10-21

**Authors:** Chen Wang, Xiaowen He, Xinxin Wang, Shuxin Zhang, Xingqi Guo

**Affiliations:** 1 State Key Laboratory of Crop Biology, College of Life Sciences, Shandong Agricultural University, PR China; 2 State Key Laboratory of Crop Biology, Shandong Agricultural University, PR China

**Keywords:** Cotton, feedback loop, *Fusarium oxysporum*, *GhMKK6*, ghr-miR5272a, *Gossypium hirsutum*, miRNA

## Abstract

Fusarium wilt is a major biotic stress affecting the productivity of cotton (*Gossypium hirsutum*). Although mitogen-activated protein kinase (MAPK) cascades play critical roles in plant disease resistance, their intricate regulation under fungal stress remains unclear, especially with regards to microRNA-mediated regulation of MAPK gene expression. In this study, we report that the MAPK kinase gene *GhMKK6* and ghr-miR5272a work together in cotton resistance to Fusarium wilt. Silencing *GhMKK6* in cotton decreased resistance to *F. oxysporum* by repressing the expression of known disease-resistance genes. Furthermore, although *GhMKK6* played a positive role in disease resistance, excessive GhMKK6 activation caused an excessive hypersensitive response. ghr-miR5272a, a major regulator, prevents this excessive response by regulating *GhMKK6* expression. ghr-miR5272a targets the *GhMKK6* 3′-untranslated region in cotton. Overexpressing miR5272a decreased the expression of *GhMKK6* and disease-resistance genes, and increased sensitivity to *F. oxysporum*, yielding a similar phenotype to *GhMKK6*-silenced cotton. Overall, these results demonstrate that the ghr-miR5272a-mediated regulation of *GhMKK6* expression contributes to the immune response in cotton, and reveal a new feedback loop mechanism in plant disease response.

## Teaser

*GhMKK6* and ghr-miR5272a comprise a feedback loop in cotton and ghr-miR5272a-mediated regulation of *GhMKK6* expression contributes to the immune response against Fusarium wilt.

## Introduction

Cotton (*Gossypium hirsutum*) is one of the most important economic crops in the world, but productivity is constrained by various biotic and abiotic stresses (Xie *et al.*, 2015). Fungal diseases such as Fusarium and Verticillium wilt pose the largest threat to cotton (Zhang *et al.*, 2016). These pathogens deliver effector molecules into plant cells to promote virulence and cause disease (Dangl and Jones, 2001). In particular, Fusarium wilt, which is caused by the phytopathogenic fungus *Fusarium oxysporum* f.sp. *vasinfectum*, is considered one of the most serious factors affecting yield and quality loss and is present in almost all cotton-growing regions worldwide (Gaspar *et al.*, 2014). Two methods have traditionally been used against the pathogen: biological control measures, such as cultivar choice and crop rotation, and chemical control (Zhang *et al.*, 2016). However, effective methods for controlling *F. oxysporum* infection are still lacking. The use of transgenic technology to control the cotton bollworm *Helicoverpa armigera* offers an alternative approach to enhance plant resistance to fungal pathogens. The mining of key resistance genes is considered the most effective method for developing pathogen-resistant varieties (Xu *et al.*, 2016), and hence studies of cotton resistance genes and the molecular mechanism of disease resistance is very important.

Mitogen-activated protein kinase (MAPK) cascades are present in all eukaryotic organisms and function in succession to transmit a variety of cellular signals (Widmann *et al.*, 1999). Activation of MAPK cascades is a common mechanism for regulating innate immune responses in both animals and plants (Pedley and Martin, 2005; Rodriguez *et al.*, 2010). A canonical MAPK cascade includes three tiered protein kinase modules: MAPK kinase kinase (MAPKKK) phosphorylates MAPK kinase (MKK), which in turn phosphorylates and activates downstream MAPKs (MAPK Group, 2002). MKKs are of particular importance (Jia *et al.*, 2016), serving as key nodes for the convergence and divergence of signals in MAPK cascades. In Arabidopsis, MAPKKK1, MKK1/MKK2, and MAPK4 function together in a MAPK cascade to regulate innate immunity (Gao *et al.*, 2008). *mkk1*/*mkk2* mutant seedlings accumulate high levels of H_2_O_2_, display spontaneous cell death, constitutively express pathogenesis-related (PR) genes, and exhibit pathogen resistance (Gao *et al.*, 2008; Pitzschke *et al.*, 2009; Kong *et al.*, 2012). The MKK3-mediated pathogen signalling pathway enhances resistance against *Pseudomonas syringae* pv. *tomato* DC3000 by positively regulating the expression of PR genes (Dóczi *et al.*, 2007). AtMKK4/AtMKK5, which can activate the MAPKs MPK3 and MPK6, plays important roles in flagellin perception and innate immunity (Asai *et al.*, 2002). Zhang *et al.* (2007) reported that *AtMKK7* positively regulates plant basal and systemic acquired resistance (SAR) and that silencing *MKK7* using antisense RNA not only compromises basal resistance but also blocks the induction of SAR. In contrast to model plants, our understanding of the function of MKKs under biotic stress in cotton is limited. In our previous studies, we have demonstrated that two group-C MKK genes, *MKK4* and *MKK5*, are involved in cotton disease resistance. Overexpression of *MKK4* enhanced sensitivity to bacterial and fungal pathogens (Li *et al.*, 2014), while overexpression of *MKK5* in *Nicotiana benthamiana* induced PR gene expression and enhanced resistance to the bacterial pathogen *Ralstonia solanacearum* (Zhang *et al.*, 2012). These data collectively suggest that MKKs play an important role in plant disease resistance. However, the function of group-A MKK genes under fungal stress in cotton remains unclear.

MAPKs generally phosphorylate their target proteins, including enzymes or transcription factors, to control the synthesis of defence hormones and signalling molecules (Bolouri Moghaddam *et al.*, 2016). However, few studies have examined the regulation of MAPK gene expression. In recent years, many MAPK genes have been predicted as targets of microRNAs (miRNAs), which are a class of small (18–24 nt), endogenous, non-coding RNAs (Bartel, 2009). miRNAs are widely distributed in plants and animals and negatively regulate gene expression at the transcriptional, post-transcriptional, and translational levels by targeting mRNAs for degradation and/or by repressing translation (Mallory and Vaucheret, 2004; Lanet *et al.*, 2009; Chellappan *et al.*, 2010). The role of miRNAs as transcriptional regulators in plant disease resistance has received increasing attention (Dugas and Bartel, 2004). High-throughput sequencing has identified many plant miRNA families that participate in the response to *Pseudomonas syringae* infection, including miR156, miR159, miR172, and miR393 (Zhang *et al.*, 2011). The first evidence of a role for miRNAs in plant immunity was the miR393-mediated repression of auxin signalling in bacterial resistance (Navarro *et al.*, 2006). In cotton, Yin *et al.* (2012) identified 215 miRNA families by high-throughput sequencing, including 14 new miRNAs. In addition, they found that the expression of 65 of the miRNA families changed dramatically after infection by *Verticillium dahlia*. miR482 and its target nucleotide binding site–leucine-rich repeat (NBS-LRR) defence genes play important roles in cotton disease resistance, and miR482 expression is inhibited and the expression of NBS-LRR genes is induced after *V. dahlia* infection (Zhu *et al.*, 2013). Although MKKs and miRNAs play important roles in cotton disease resistance, little is currently known about the functions and relationships between them.

In this study, a new cotton group-A MKK gene, *GhMKK6*, was identified and found to be indispensable for the response to *F. oxysporum*. However, excessive *GhMKK6* activation can lead to hypersensitive response-like cell death and give rise to lesion-mimicking phenotypes. The results from a series of genomic, genetic, transgenic, and virus-induced gene-silencing (VIGS) experiments suggest that the ghr-miR5272a-mediated control of *GhMKK6* gene expression contributes to the immune response. Our findings provide important information regarding the relationship between MKKs and miRNAs in the immune response in cotton.

## Materials and methods

### Plant material, growth conditions, and stress treatments

Seeds of cotton (*Gossypium hirsutum* L. cv. lumian 22) were germinated in wet linen. Germinated seedlings were then transplanted into hydroponic cultures under greenhouse conditions at 28 ± 1 °C with a 16-h light/8-h dark cycle and 60–75% relative humidity. For salicylic acid (SA) (10 mM) and methyl jasmonate (MeJA) (100 μM) treatments, cotyledons were sprayed with each chemical. For the pathogen treatment, seedlings were inoculated with conidial *Fusarium oxysporum* suspensions (10^6^ conidia ml^–1^) using the root-dip method. For expression pattern analyses, cotyledons from treated plants were collected, frozen in liquid nitrogen, and stored at –80 °C for RNA extraction. For *F. oxysporum* treatments that were performed on *GhMKK6*-silenced or amiR5272-overexpressing cotton, the plants were inoculated with conidial *F. oxysporum* suspensions (10^6^ conidia ml^–1^) using the root-dip method. *Nicotiana benthamiana* seeds were surface-sterilized and germinated on Murashige–Skoog (MS) medium under greenhouse conditions at 25 ± 1 °C with a 16-h light/8-h dark cycle and 60–75% relative humidity. The seedlings were then transplanted at the 2–3-leaf stage into soil and grown under greenhouse conditions. For *F. oxysporum* treatments that were performed on *GhMKK6*-overexpressing tobacco, the leaves were injected with 100 μl of conidial *F. oxysporum* suspensions (10^6^ conidia ml^–1^) using syringes. Each treatment was repeated at least three times.

### Gene cloning and bioinformatics analysis

The *GhMKK6* gene was isolated from a cotton cDNA library via PCR (primers are listed in Supplementary Table S1 at *JXB* online). GhMKK6-homologous protein sequences were retrieved from NCBI (http://blast.ncbi.nlm.nih.gov/Blast.cgi) and aligned using DNAMAN 5.2.2 software (Lynnon Biosoft). The dendrogram was generated using the neighbour-joining method in MEGA 5.0 software.

### RNA extraction and qRT-PCR analysis

Total RNA was isolated from cotton seedlings and tobacco leaves using the CTAB method (Wang *et al.*, 2011) and the TRIzol reagent (Takara, Japan), respectively. First-strand cDNA was synthesized using an EasyScript First-Strand cDNA Synthesis SuperMix kit (TransGen Biotech, Beijing, China). For miRNA qRT-PCR, first-strand cDNA was synthesized using a Mir-X^TM^ miRNA First Strand Synthesis Kit (Takara, Japan). The miRNA-specific primer was designed based on the mature miRNA sequence (see Supplementary Table S1). qRT-PCR was performed using SYBR Premix Ex Taq (Takara) in a 20-μl reaction volume on a CFX96^TM^ Real-time Detection System (Bio-Rad). The PCR program was as follows: pre-denaturation at 95 °C for 30 s; 40 cycles of 95 °C for 30 s, 55 °C for 15 s and 72 °C for 15 s; and a melt cycle from 65 to 95 °C. The 2^−ΔΔ*C*T^ method was used to determine the relative expression levels. The *UBI* and *β-actin* genes from *G. hirsutum* and *N. benthamiana*, respectively, were used as standard controls. The primers used for qRT-PCR are listed in Supplementary Tables S1 and S2.

### Vector construction, genetic transformation, and site-directed mutagenesis

The *GhMKK6* ORF was cloned and inserted into the pRI 201-AN-GUS vector (Takara, Japan). The genetic transformation was performed as described previously by Lu *et al.* (2013). The T_3_ progeny of the empty vector or transgenic plants were used for further functional studies.

A constitutively active mutant of *GhMKK6*, GhMKK6EE, was obtained as described previously by replacing the conserved Ser-219 and Thr-225 with Glu (Asai *et al.*, 2008). The inactive *GhMKK6* mutant, GhMKK6AA, in which the conserved Ser-219 and Thr-225 were replaced with Ala, was obtained via the same method. The activities of GhMKK6EE and GhMKK6AA have been tested (see Supplementary Fig. S7). The primers used for mutagenesis are shown in Supplementary Table S1.

### ghr-miR5272a overexpression and suppression assays and virus-induced gene silencing (VIGS)

The method was performed as described by Gu *et al.* (2014). Two *GhMKK6* fragments (nucleotides 451–934 and nucleotides 136–522), a small tandem target mimic sequence (containing two imperfect ghr-miR5272a binding sites separated by a 48-bp spacer; see Supplementary Fig. S11A), and the full-length amiR5272 precursor (ath-miR319a replaced with the mature ghr-miR5272a sequence) were inserted into pCLCrVA to produce pCLCrVA-*GhMKK6*, pCLCrVA-STTM, and pCLCrVA-amiR5272, respectively. The primers used are shown in Supplementary Table S1. The pCLCrVA, pCLCrVB, and recombinant plasmids were transformed into *Agrobacterium tumefaciens* strain EHA105. Cultures were grown overnight at 28 °C in lysogeny broth (LB) medium containing 50 μg ml^–1^ kanamycin and 50 μg ml^–1^ rifampicin, pelleted, and individually resuspended (OD_600_=1) in infiltration media (10 mM MgCl_2_, 10 mM MES-NaOH, and 200 μM acetosyringone). Following incubation for 3 h, a mixture of equal parts *Agrobacterium* suspension containing pCLCrVA or pCLCrVB was inoculated into two fully expanded cotton cotyledons. The leaves from the inoculated seedlings were used for assays 3 weeks after inoculation. Each assay was performed with at least three independent replicates.

### *Agrobacterium*-mediated transient expression in *N. benthamiana*

A *GhMKK6*, *GhMKK6EE*, or *GhMKK6AA* fragment was inserted into the pRI 201-AN-GUS vector (Takara, Japan) and transformed into *A. tumefaciens* strain GV3101. Overnight cultures were then pelleted and individually resuspended (OD_600_=1) in infiltration media as described above. After 3 h of incubation, the suspensions were introduced into *N. benthamiana* leaves.

For tobacco leaf co-transformation assays using ghr-miR5272a and *GhMKK6*, a *GhMKK6* fragment (containing the *GhMKK6* ORF or the *GhMKK6* ORF and 3′-UTR) was PCR-amplified and inserted into pRI 201-AN-GUS (Takara, Japan). amiR5272 was inserted into pRI 201-AN (Takara, Japan). The primers used for making the plasmids are listed in Supplementary Table S1. The recombinant vectors were transformed into *A. tumefaciens* strain GV3101. The transient expression method was as described above. Each assay was performed with at least three independent replicates.

### MAPK activation assays

Proteins were extracted from leaves using extraction buffer [50 mM Tris/HCl (pH 7.5), 150 mM NaCl, 50 mM EDTA, 1% (v/v) Triton X-100, 0.1% (w/v) SDS, phosphatase and protease inhibitors (PhosStop™ and EDTA-free Complete™, Roche)]. MAPK activation assays were performed as described previously by Ovečka *et al.* (2014). The anti-pTEpY phospho-p44/42 MAPK antibody (Cell Signaling Technology, USA) was used to detect the activation of MAPKs.

### Pathogen biomass assays

DNA was isolated using the DNAsecure Plant Kit (Tiangen, China), and the amounts of *F. oxysporum* (cutinase) DNA relative to cotton (GhSK11) or tobacco (NtSK11) DNA were determined by qPCR as described previously (Gachon and Saindrenan, 2004; Tsuda *et al.*, 2009). Amplification reactions were performed using SYBR Premix Ex Taq (TaKaRa, Japan) on a CFX96^TM^ Real-time Detection System (Bio-Rad). Disease index [log_2_ (CutA/SK11)] values were obtained by subtracting the *C*_t_ values of the cutinase from those of SK11 (Tsuda *et al.*, 2009). Three biological replicates were analysed for all of the samples.

### Histochemical staining assay

For 3'-diaminobenzidine (DAB) staining, leaves were soaked in a solution containing 1 mg ml^–1^ DAB (pH 3.8) in the dark for 12 h. Next, chlorophyll was removed from the leaves using 95% ethanol. Trypan blue staining was performed as described previously by Zhang *et al.* (2012). β-Glucuronidase (GUS) staining was carried out as described by Jefferson *et al.* (1987).

### Statistical analysis

All experiments were performed at least three times. Statistically significant differences between measurements were determined using the Tukey HSD test in IBM Statistical Product and Service Solutions (SPSS) Statistics software version 19 (IBM, USA).

### Accession numbers

The *GhMKK6* sequence can be found at http://cottongen.org under the Unique Name CotAD_25213_BGI-AD1_v1.0. Sequence data for the other cotton genes discussed in this paper can be found at http://cottongen.org under the following Unique Names: GhMKK5 (CotAD_01560_BGI-AD1_v1.0), GhNPR1 (CotAD_22990_BGI-AD1_v1.0), GhICS1 (CotAD_50284_BGI-AD1_v1.0), GhEDS1 (CotAD_31858_BGI-AD1_v1.0), GhPAD4 (CotAD_57749_BGI-AD1_v1.0), GhJAZ1 (CotAD_21952_BGI-AD1_v1.0), GhJAZ3 (CotAD_67052_BGI-AD1_v1.0), GhAOS (CotAD_73823_BGI-AD1_v1.0), GhAOC4 (CotAD_21497_BGI-AD1_v1.0), GhRbohB (CotAD_38218_BGI-AD1_v1.0), and GhSK11 (CotAD_53331_BGI-AD1_v1.0). Sequence data regarding the Arabidopsis genes discussed in this paper can be found in the TAIR database (http://arabidopsis.org) under the following accession numbers: AtMKK1 (AT4G26070), AtMKK2 (AT4G29810), AtMKK6 (AT5G56580), AtMKK3 (AT5G40440), AtMKK4 (AT1G51660), AtMKK5 (AT3G21220), and AtMKK7 (AT1G18350). Sequence data for the other genes can be found in the GenBank database (https://ncbi.nlm.nih.gov/genbank/) under the following accession numbers: FoCut (EXM24724), NtMEK1 (NP_001312945), NtNPK2 (BAA06731), NtMEK2 (NP_001312017), NtNPR1 (KY402167), NtICS1 (XM_016616488), NtEDS1 (XM_016636310), NtPAD4 (XM_016623921), NtJAZ1 (GU066894), NtJAZ3 (AB433898), NtAOS (AB778304), NtAOC4 (XM_019406620), NtrbohB (XM_019378071), NtSK11 (NM_001325321), ZmMKK1 (AAK73104), CsMKK8 (XP_010489696), and BrMKK10 (XP_009114895).

## Results

### GhMKK6 is indispensable for cotton resistance to *Fusarium oxysporum*

**Fig. 1.**
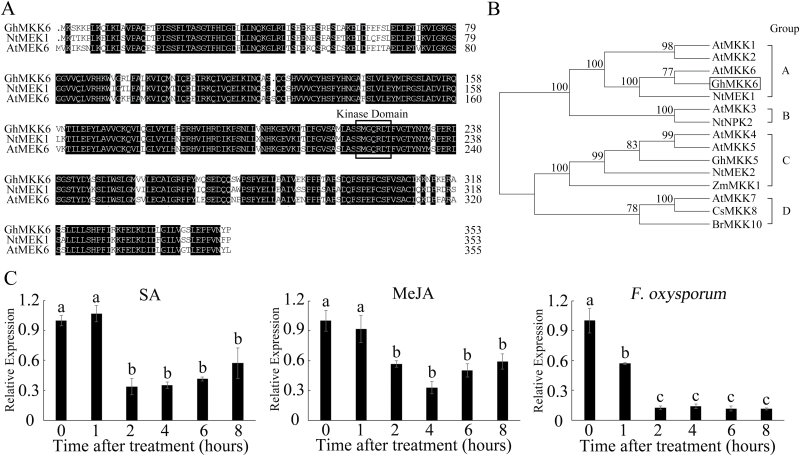
Characterization and sequence analysis of *GhMKK6*. (A) Multiple amino acid sequence alignment of GhMKK6, AtMEK6, and NtMEK1. The conserved kinase domain S/TXXXXXS/T is indicated by the box. (B) Dendrogram analysis. The numbers above the branches indicate bootstrap values (>50%) from 1000 replicates. A, B, C, and D indicate MAPKK groups. (C) qRT-PCR analysis of *GhMKK6* expression under SA, MeJA, or *F. oxysporum* treatment. Data are means ± SE of three independent experiments (*n*=6). Different letters indicate significant differences (*P*<0.01) based on Tukey’s HSD test.

Previous studies have reported that the MKK6-mediated MAPK cascade pathway plays important roles in plant growth and development, production of reactive oxygen species (ROS), and disease resistance in many model plants (Asai *et al.*, 2008; Kosetsu *et al.*, 2010; Deng *et al.*, 2016). MKK6 is an important component and a key regulator of this cascade. To determine the functions of the *MKK6* gene in cotton, a cDNA sequence that displayed high similarity to *AtMKK6* and *NtMEK1* was identified. The full-length *GhMKK6* cDNA consisted of a 50-bp 5′-UTR, a 158-bp 3′-UTR, and a 1065-bp ORF. The ORF encodes a 354-amino-acid protein with a calculated molecular mass of 39.868 kDa and an isoelectric point of 6.73. Sequence analysis of GhMKK6 revealed strong homology with AtMKK6 (88.20%) and NtMEK1 (88.42%) (Fig. 1A). The deduced amino acid sequence of *GhMKK6* was found to contain a kinase domain (S/TXXXXXS/T) from amino acids 219 to 225 (Fig. 1A). Further dendrogram analysis indicated that GhMKK6 belongs to the group-A MKKs and is closest to AtMKK6 and NtMEK1 (Fig. 1B). Next, the pattern of *GhMKK6* expression was determined using qRT-PCR. *GhMKK6* was expressed in all tissues investigated in this study, particularly in the roots (Supplementary Fig. S1). As shown in Fig. 1C, *GhMKK6* expression was reduced by phytohormones such as SA and MeJA as well as by *F. oxysporum* treatment.

**Fig. 2.**
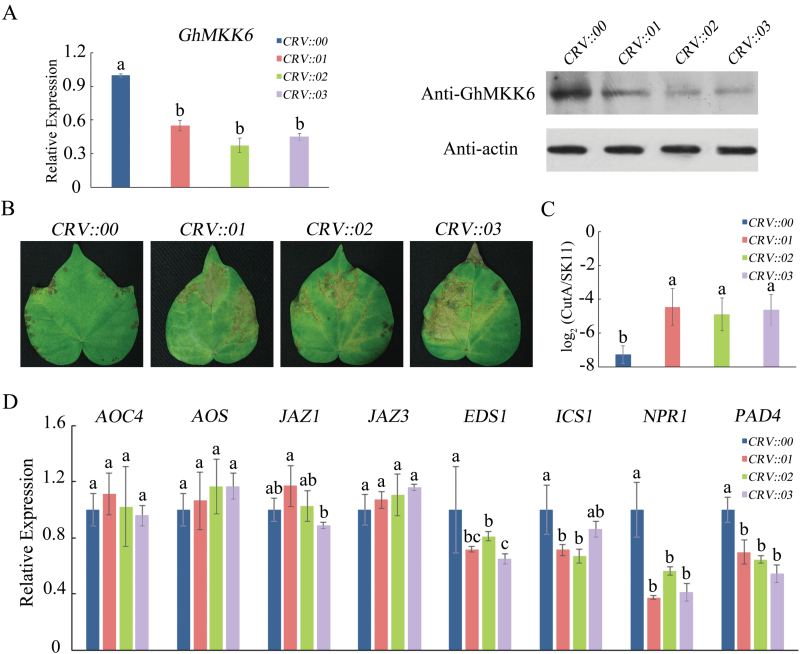
*GhMKK6*-silencing in cotton reduces resistance to *F. oxysporum*. (A) *GhMKK6* RNA relative expression and protein levels in *GhMKK6*-silenced cotton. (B) Representative phenotypes of *GhMKK6*-silenced plants 5 d after being infected with *F. oxysporum*. (C) Pathogen disease index in *GhMKK6*-silenced plants 5 d after *F. oxysporum* infection. (D) qRT-PCR analysis for expression of SA- and JA-mediated defence pathway genes in *CRV::00*, *CRV::01*, *CRV::02*, and *CRV::03* at 5 d after infection with *F. oxysporum*. *CRV::00* served as the empty vector control. Data in (A, C, D) are means ± SE of three independent experiments; (A, D) *n*=15, (C) *n*=6. Different letters indicate significant differences (*P*<0.01) based on Tukey’s HSD test. (This figure is available in colour at *JXB* online.)

To investigate the biological role of *GhMKK6*, *Agrobacterium*-mediated VIGS was used to silence *GhMKK6* in cotton. Three weeks after *Agrobacterium* infiltration, *GhMKK6* RNA and protein levels were significantly reduced (the region of *GhMKK6* used for silencing was nucleotides 451–934) (Fig. 2A). To examine the role of *GhMKK6* in the defence response to *F. oxysporum*, *CRV::00* (empty vector control) and *CRV::GhMKK6* (*CRV::01*, *CRV::02*, and *CRV::03*) were root-wounded and placed in a *F. oxysporum* spore suspension using the root-dip method. At 5 d after inoculation, *CRV::GhMKK6* leaves showed serious signs of chlorosis, whereas *CRV::00* leaves exhibited only mild disease (Fig. 2B). The pathogen disease index also showed that *CRV::GhMKK6* leaves accumulated more *F. oxysporum* than those from *CRV::00* (Fig. 2C). We then analysed the expression patterns of several genes in the SA- or JA-mediated defence pathways. As shown in Fig. 2D, the expression levels of SA-mediated genes (*EDS1*, *ICS1*, *NPR1*, and *PAD4*) were obviously lower in *CRV::GhMKK6* leaves than in *CRV::00* leaves. In contrast, the expression levels of JA-mediated genes (*AOC4*, *AOS*, *JAZ1*, and *JAZ3*) did not differ significantly between *CRV::GhMKK6* and *CRV::00* leaves. Because the MAPK cascade pathway can regulate both early and long-term plant stress responses, the expression of SA- and JA-mediated defence-pathway genes was also determined at 4 h and 12 h after *F. oxysporum* inoculation (see Supplementary Fig. S2A, B). At 4 h after *F. oxysporum* inoculation, the expression of SA-mediated genes exhibited no obvious changes (Fig. S2A), and only the expression of *NPR1* was reduced in *CRV::GhMKK6* leaves at 12 h after inoculation (Fig. S2B). To prove the validity of our data, we used VIGS to silence *GhMKK6* using another fragment from nucleotides 136–522 (*CRV::01-2* and *CRV::02-2*) (Supplementary Fig. S3B). The expression levels of SA-mediated genes were lower in *CRV::01-2* and *CRV::02-2* than those in *CRV::00*, and the expression levels of JA-mediated genes did not differ significantly (Fig. S3C). These results indicated that *GhMKK6* may be associated with the SA-mediated defence pathway and play an important role in fungal defence in cotton.

### Excessive GhMKK6 activation leads to lesion-mimicking phenotypes

In recent studies, silencing *MEK1* (the gene homologous to *GhMKK6*) in tobacco was found to decrease resistance to pathogens (Asai *et al.*, 2008; Liu *et al.*, 2004), and we noticed that silencing *GhMKK6* in cotton produced similar phenotypes to those observed after pathogen infection. To determine the function of *GhMKK6* in plant disease resistance, we overexpressed *GhMKK6* in *N. benthamiana*. Three independent lines were selected for further functional analysis (see Supplementary Fig. S4).

**Fig. 3.**
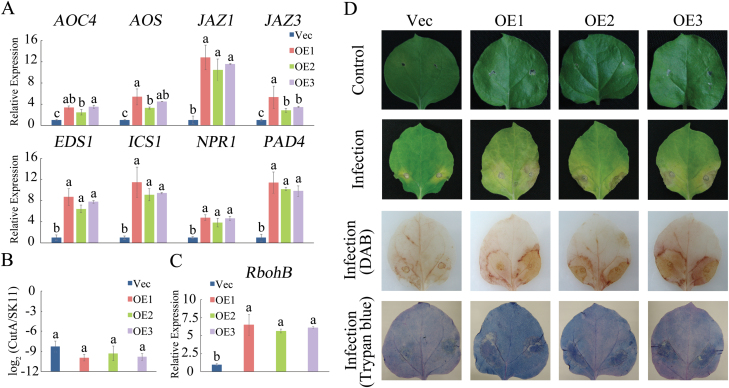
Phenotypes of *GhMKK6*-overexpressing plants infected with *F. oxysporum*. (A) qRT-PCR analysis of expression of SA- and JA-mediated defence pathway genes in *GhMKK6*-overexpressing plants at 3 d after *F. oxysporum* infection. (B) Pathogen disease index in *GhMKK6*-overexpressing plants at 3 d after *F. oxysporum* infection. (C) qRT-PCR analysis of *RbohB* expression in *GhMKK6*-overexpressing plants at 3 d after *F. oxysporum* infection. Data in (A–C) are means ± SE of three independent experiments (*n*=6). Different letters indicate significant differences (*P*<0.05) based on Tukey’s HSD test. (D) Representative phenotypes of empty vector (Vec) and *GhMKK6*-overexpressing (OE) plants infected with *F. oxysporum* (at 3 d after infection). DAB staining was used to show ROS accumulation, and Trypan blue staining was used to show the level of cell death. (This figure is available in colour at *JXB* online.)

To confirm the resistance of *GhMKK6*-overexpressing (OE) plants to *F. oxysporum*, we examined 6-week-old empty vector (Vec) and transgenic lines. At 3 d after *F. oxysporum* inoculation, the MAPK phosphorylation levels in transgenic plants were much higher than those in Vec plants (see Supplementary Fig. S5). At 4 h and at 3 d after *F. oxysporum* inoculation, changes were detected in the expression patterns of several genes in the SA- and JA-mediated defence pathways. The expression levels of the selected genes in the OE lines were higher than those in the Vec plants (shown at 4 h in Supplementary Fig. S6 and at 3 d in Fig. 3A). Although pathogen growth assays showed that the *F. oxysporum* disease indices in leaves from OE lines and Vec plants were not obviously different (Fig. 3B), the OE lines showed serious signs of chlorosis (Fig. 3D). Histochemical staining with Trypan blue (depicting cell death) and DAB (depicting ROS accumulation) showed that OE leaves accumulated more of the stains than the Vec leaves (Fig. 3D).

**Fig. 4.**
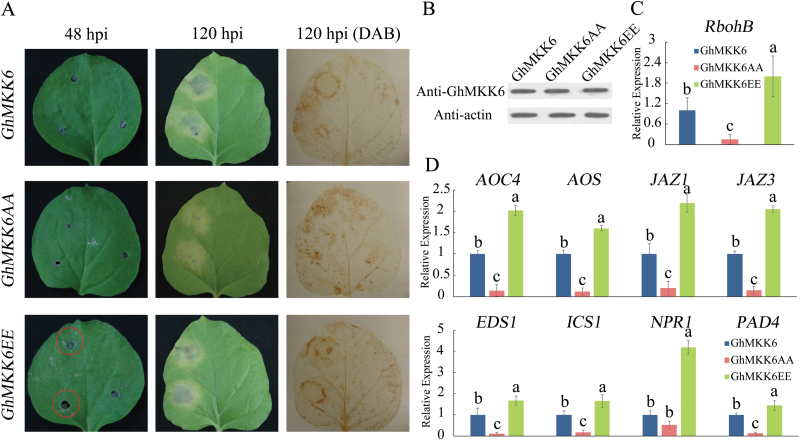
Excessive GhMKK6 activation leads to lesion-mimicking phenotypes. (A) Representative phenotypes of plants transiently expressing *GhMKK6*, *GhMKK6AA*, and *GhMKK6EE* at 48 and 120 h post-infiltration (hpi). DAB staining was used to show ROS accumulation. (B) GhMKK6 protein levels in plants transiently expressing *GhMKK6*, *GhMKK6AA*, and *GhMKK6EE* at 5 d post-infiltration. (C, D) qRT-PCR analysis of expression of (C) *rbohB* and (D) SA- and JA-mediated defence pathway genes in plants transiently expressing *GhMKK6*, *GhMKK6AA*, and *GhMKK6EE* at 5 d post-infiltration. Data are means ± SE of three independent experiments (*n*=6). Different letters indicate significant differences (*P*<0.05) based on Tukey’s HSD test. (This figure is available in colour at *JXB* online.)

Based on the above results and previous studies, we speculated that excessive GhMKK6 activation causes lesion-mimicking phenotypes in *GhMKK6*-overexpressing lines after *F. oxysporum* inoculation. To confirm our hypothesis, we constructed a constitutively active *GhMKK6* mutant, GhMKK6EE, to simulate activated GhMKK6, and an inactive version of *GhMKK6*, GhMKK6AA, and expressed each under the control of the CaMV 35S promoter (see Supplemntary Fig. S7A). Then, *GhMKK6*, *GhMKK6EE*, and *GhMKK6AA* were expressed transiently by *Agrobacterium*-infiltration in *N. benthamiana* (Fig. 4B). At 5 d after infiltration (=120 h post-infiltration, hpi), leaves expressing *GhMKK6EE* showed the lesion-mimicking phenotype (Fig. 4A). The expression patterns of SA- and JA-mediated defence-pathway genes were also examined. As shown in Fig. 4D, while the expression levels of those genes in *GhMKK6* plants were normal, they were remarkably higher in *GhMKK6EE* leaves than in *GhMKK6* or *GhMKK6AA* leaves. The respiratory burst oxidase homolog protein B (RbohB) plays important roles in ROS production, and *RbohB* expression levels in OE- and *GhMKK6EE*-expressing leaves were increased (Fig. 3C and 4C). At 3 d after *N. benthamiana* leaves were infected with *GhMKK6*, *GhMKK6EE*, and *GhMKK6AA*, detached leaves were placed in an *F. oxysporum* spore suspension (10^6^ conidia ml^–1^). Although all of the leaves exhibited signs of chlorosis, the pathogen growth assay showed that leaves expressing *GhMKK6EE* accumulated less *F. oxysporum* than those expressing *GhMKK6AA* (Supplementary Fig. S7B, C). Thus, we confirmed that excessive GhMKK6 activation engendered a lesion-mimicking phenotype.

### GhMKK6 is a target of ghr-miR5272a

**Fig. 5.**
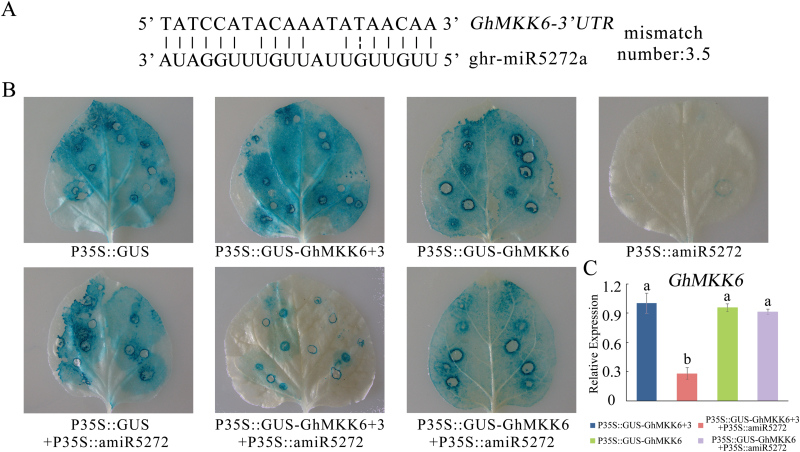
*GhMKK6* is a ghr-miR5272a target. (A) Prediction and validation of ghr-miR5272a targets. (B) Co-transformation of tobacco leaves with *GhMKK6 + 3*, *GhMKK6*, and amiR5272. GUS staining was used to show the levels of transcripts. (C) *GhMKK6* expression levels in different co-transformed tobacco leaves. Data are means ± SE of three independent experiments (*n*=9). Different letters indicate significant differences (*P*<0.01) based on Tukey’s HSD test. (This figure is available in colour at *JXB* online.)

Based on these results, we deduced that although *GhMKK6* played a positive role in cotton disease resistance, excessive GhMKK6 activation could be harmful. From the expression pattern assays, we speculated that the transcriptional and/or post-transcriptional regulation of *GhMKK6* may be used to avoid excessive GhMKK6 activation. miRNAs are ubiquitous transcriptional and/or post-transcriptional regulators in plants, and previous studies have shown that they play important roles in cotton disease resistance (He *et al.*, 2014). By analysing high-throughput sequencing data from previous studies, we found that ghr-miR5272a matched the *GhMKK6* 3′-UTR (Xie *et al.*, 2015). In nature, a functional miRNA and its target can contain up to five mismatches (Schwab *et al.*, 2005); our analysis revealed 3.5 mismatches within the predicted complementary region between ghr-miR5272a and *GhMKK6*, with G:U pairs counting as 0.5 mismatches (Fig. 5A). This result indicated that ghr-miR5272a might cleave the *GhMKK6* transcript.

To investigate whether ghr-miR5272a cleaves *GhMKK6* mRNAs, we expressed ghr-miR5272a using the precursor of ath-miR319a as a backbone (Gu *et al.*, 2014). We exchanged the native ath-miR319a sequence with ghr-miR5272a, yielding amiR5272, which was then inserted into the pRI 201-AN vector (the GUS gene was removed). *Agrobacterium*-mediated transient expression was used to analyse the cleavage of *GhMKK6* mRNA by amiR5272. Leaves transformed with pRI 201-AN-GUS (harbouring the GUS gene) were used as a control. As shown in Fig. 5B, leaves transiently expressing 35S::GUS, 35S::GUS-GhMKK6 + 3 (containing the *GhMKK6* ORF and 3′-UTR), and 35S::GUS-GhMKK6 (only containing the *GhMKK6* ORF) showed similar GUS levels. In contrast, leaves inoculated with 35S::amiR5272 showed no GUS signal. When 35S::GUS-GhMKK6 + 3 was co-transformed into leaves with 35S::amiR5272, only very weak GUS staining was detected compared with leaves that only expressed 35S::GUS-GhMKK6 + 3. Meanwhile, leaves inoculated with both 35S::GUS-GhMKK6 and 35S::amiR5272 showed no obvious change in GUS staining compared with leaves that only expressed 35S::GUS-GhMKK6. The levels of *GhMKK6* expression in the transient leaves were also analysed. As shown in Fig. 5C, *GhMKK6* mRNA levels were reduced in leaves co-transformed with 35S::GUS-GhMKK6 + 3 and 35S::amiR5272. The protein level of GhMKK6 was also obviously reduced when ghr-miR5272a was co-expressed with GUS::GhMKK6 + 3 but not with GUS::GhMKK6 (see Supplementary Fig. S8). In *N. benthamiana*, *NbMEK1* cannot be targeted by ghr-miR5272a due to the limited complementarity of the sequence (Supplementary Fig. S9A). Ectopic expression of ghr-miR5272a in *N. benthamiana* leaves did not obviously alter *NbMEK1* expression (Fig. S9B). This result shows that ghr-miR5272a targets *GhMKK6*.

### ghr-miR5272a characterization and expression patterns in cotton

**Fig. 6.**
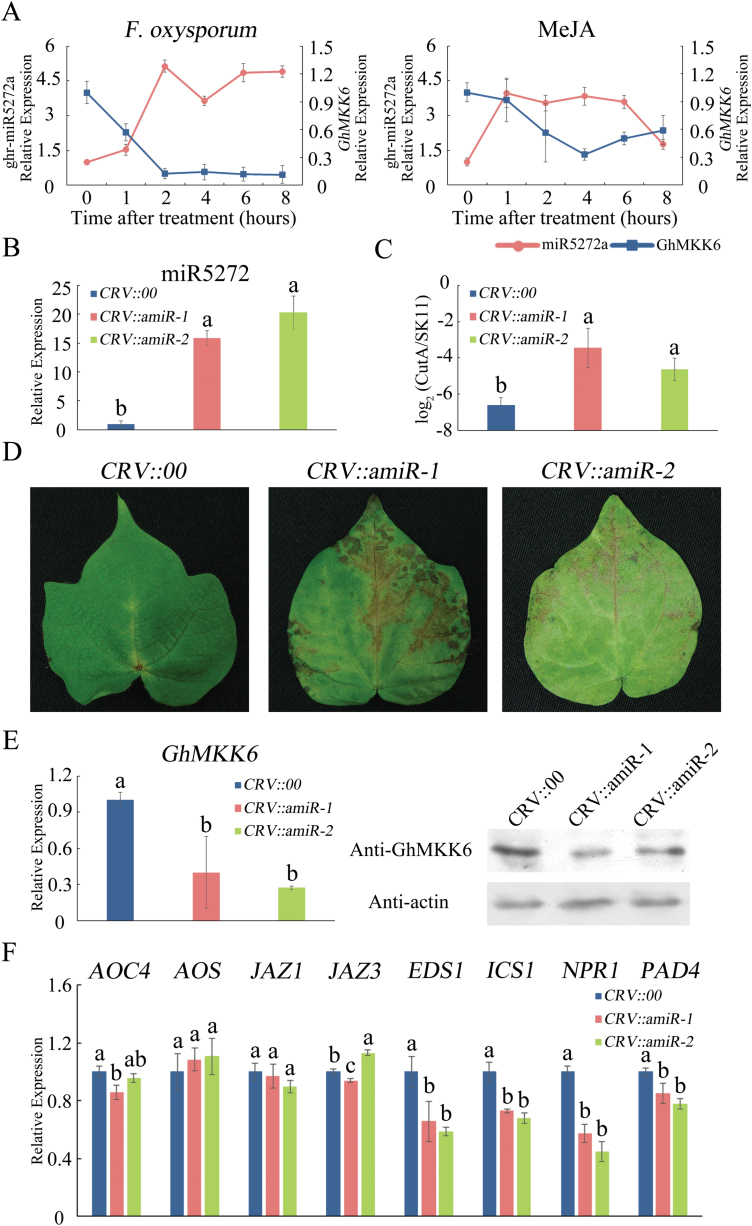
ghr-miR5272a overexpression sensitizes cotton to *F. oxysporum*. (A) ghr-miR5272a expression patterns in cotton under MeJA and *F. oxysporum* treatments. (B) miR5272 expression levels in cotton transfected with pCLCrVA-amiR5272. (C) Pathogen disease index in amiR5272-overexpressing plants at 5 d after *F. oxysporum* infection. (D) Representative phenotypes of amiR5272-overexpressing plants infected with *F. oxysporum* (5 d after infection). (E) GhMKK6 mRNA relative expression and protein levels in cotton transfected with pCLCrVA-amiR5272. (F) qRT-PCR analysis of expression of SA- and JA-mediated defence pathway genes in amiR5272-overexpressing plants at 5 d after *F. oxysporum* infection. *CRV::00* served as the empty vector control. Data in (A–C, E, F) are means ± SE of three independent experiments (*n*=6). Different letters indicate significant differences (*P*<0.05) based on Tukey’s HSD test. (This figure is available in colour at *JXB* online.)

We determined ghr-miR5272a expression after SA, MeJA, or *F. oxysporum* treatment to see if a response could be detected. As shown in Fig. 6A, ghr-miR5272a expression peaked at 1 h and decreased after 6 h following MeJA treatment. Treatment with *F. oxysporum* also obviously induced ghr-miR5272a expression (Fig. 6A). However, no significant changes in ghr-miR5272a expression were detected following SA treatment (see Supplementary Fig. S10). Thus, ghr-miR5272a expression behaved reciprocally to that of *GhMKK6* upon treatment with MeJA or *F. oxysporum* (Fig. 6A). These results indicated that *GhMKK6* is transcriptionally and/or post-transcriptionally regulated by ghr-miR5272a *in vivo*.

### ghr-miR5272a affects the cotton immune response to *Fusarium oxysporum*

Many studies have reported that short tandem target mimics (STTMs) can be used to inhibit miRNA function (Yan *et al.*, 2012). To investigate the function of ghr-miR5272a, we constructed the pCLCrVA-STTM vector, which contains two imperfect ghr-miR5272a binding sites separated by a 48-bp spacer described by Yan *et al.* (2012) (see Supplementary Fig. S11A). At 3 weeks after *Agrobacterium* infiltration, the expression of ghr-miR5272a was reduced (Fig. S11B). Leaves from *CRV::00* and *CRV::STTM* were then placed in *F. oxysporum* spore suspensions. As shown in Fig. S11C, *GhMKK6* expression was not significantly altered in *CRV::STTM* cotton. At 5 d after inoculation, the expression of several SA- and JA-mediated defence pathway genes was detected and, as shown in Fig. S11D, the accumulation of these genes was increased.

To further investigate the function of ghr-miR5272a, we inserted the amiR5272 precursor into the pCLCrVA vector to produce pCLCrVA-amiR5272. At 3 weeks after *Agrobacterium* infiltration, qPCR and western blotting were performed to detect *GhMKK6* and ghr-miR5272a expression. As shown in Fig. 6B, miR5272a transcript abundance was significantly increased in *CRV::amiR* cotton compared with *CRV::00*. As a target of ghr-miR5272a, *GhMKK6* transcription and translation were reduced in the *CRV::amiR* lines (Fig. 6E).

To examine the resistance of *CRV::amiR* cotton to infection, *CRV::00* and *CRV::amiR* were root-wounded and inoculated with *F. oxysporum* spore suspensions using the root-dip method. At 5 d after inoculation, the *CRV::amiR* leaves showed more serious signs of chlorosis than the *CRV::00* leaves, and they also accumulated more *F. oxysporum* (Fig. 6C, D). The expression patterns of several SA- and JA-mediated defence pathway genes were also examined, and the expression levels of SA-mediated genes (*EDS1*, *ICS1*, *NPR1*, and *PAD4*) were clearly lower in *CRV::amiR* leaves than in *CRV::00* leaves (Fig. 6F). In contrast, the expression levels of JA-mediated genes (*AOC4*, *AOS*, *JAZ1*, and *JAZ3*) were unaffected. These results showed that *GhMKK6*-silenced and ghr-miR5272a-overexpressing cotton have the same expression profiles under *F. oxysporum* treatment.

## Discussion

The role of MKK6 has been widely studied in model plants. In Arabidopsis, ANPs, MKK6/ANQ (homologous to MKK6 in Arabidopsis), and MPK4 function together in a MAPK cascade that is required for cytokinesis (Krysan *et al.*, 2002; Kosetsu *et al.*, 2010), and the *mkk6*/*anq* mutant exhibits severe cytokinesis defects (Takahashi *et al.*, 2010). However, in tobacco, the MKK1/MKK2-mediated MAPK pathway plays a different role in plant immune responses. MKK1 (which is homologous to MKK6 in tobacco) -NTF6 participates in the regulation by INF1 elicitin of bursts of ROS, and constitutively expressing MEK1^DD^ induces RbohB-dependent oxidative bursts (Asai *et al.*, 2008). Liu *et al.* (2004) also reported that the MEK1-NTF6 pathway was involved in virus immunity in *N. benthamiana*; silencing MEK1 using VIGS decreased resistance to Tobacco mosaic virus. These data collectively suggest that the function of MKK6 is very complex, and hence studying it in other plants is very important to understand the molecular mechanisms of plant disease resistance. In the present study, we confirmed that *GhMKK6* also plays an important role in cotton disease resistance, and silencing *GhMKK6* decreased tolerance to Fusarium wilt and decreased the expression of several resistance genes, including *EDS1*, *PAD4*, and *ICS1* (Fig. 2). However, gain-of-function analyses showed that excessive GhMKK6 activation overbalanced SA-mediated defence responses and ROS production, and resulted in lesion-mimicking phenotypes (Fig. 3). Although the use of overexpression of *GhMKK6* in tobacco to determine its function in cotton has some limitations, our data do indicate the potential function of *GhMKK6* in cotton. Interestingly, we noticed that many genes involved in JA biosynthesis, such as *AOS* and *AOC4*, and in JA signalling, such as *JAZ1* and *JAZ3*, were up-regulated in *GhMKK6*-overexpressing lines, whereas the expression of these genes showed no significant changes in *GhMKK6*-silenced cotton (Figs. 2 and 3). These results imply that *GhMKK6* may also participate in the JA-mediated defence pathway, and other genes may play the same role in JA-mediated defence responses when *F. oxysporum* infects cotton species. Recent reports have shown that imbalanced JA signalling also causes hypersensitive response (HR)-like cell death and engenders lesion-mimicking phenotypes (Sun *et al.*, 2014). Overall, we conclude that *GhMKK6* is necessary for cotton disease resistance but that excessive GhMKK6 activation may have negative effects.

In the absence of pathogens, plant defence responses must be kept under tight control to prevent autoimmunity (Kong *et al.*, 2012). In recent years, miRNA-mediated gene expression regulation has received widespread attention. Compared with studies in model plant species, studies of cotton miRNAs are limited (Wang and Zhang, 2015). miR5272a was previously identified as a conserved miRNA family in *Medicago truncatula* (Devers *et al.*, 2011). Recently, deep sequencing identified miR5272a in cotton and revealed *GhMKK6* as a potential target (Xie *et al.*, 2015). ghr-miR5272a expression does not change significantly in response to abiotic stress (Xie *et al.*, 2015). He *et al.* (2014) reported that ghr-miR5272a may be involved in regulating Verticillium wilt resistance in cotton. They found that ghr-miR5272a expression was higher in plants infected with *V. dahlia* D07038 (an intermediately aggressive stain) than in plants infected with *V. dahlia* V991 (a highly toxic stain). In our study, we characterized the expression patterns and biological functions of ghr-miR5272a and provided evidence of ghr-miR5272a-mediated *GhMKK6* expression regulation as a new regulatory mechanism to prevent excessive autoimmunity in cotton under *F. oxysporum* infection. Due to the limitations of experimental conditions, transient transfections in cotton are very difficult. Tobacco was therefore used instead, and our results showed that ghr-miR5272a could cleave *GhMKK6* mRNA *in vivo* (Fig. 5). However, the cleavage site may not exist in *N. benthamiana MKK1*, as determined by a bioinformatics analysis, and transient expression of ghr-miR5272a in *N. benthamiana* showed no obvious effects on *MKK1* expression. Therefore, this miR5272a-mediated gene expression regulation mechanism may be limited to certain species.

**Fig. 7.**
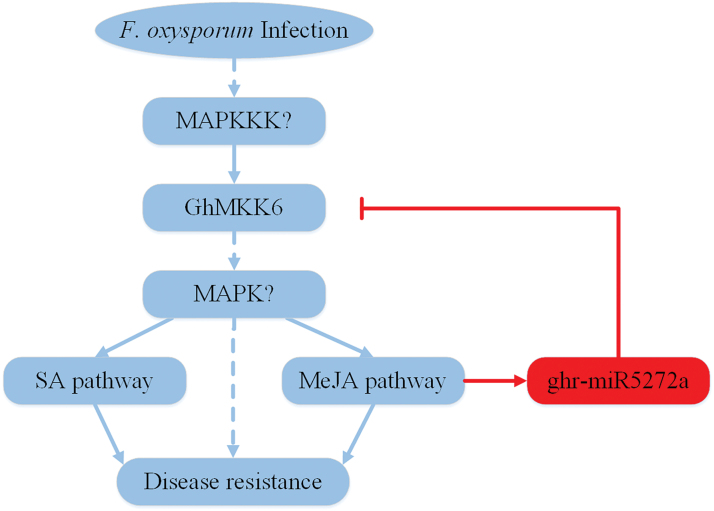
Schematic representation of the proposed model for the GhMKK6–miR5272a feedback loop. After *F. oxysporum* infects cotton, the GhMKK6-mediated signal transduction pathway is activated, which enhances *F. oxysporum* resistance via inducing SA- and/or JA-mediated defence responses. miR5272a regulates the expression of *GhMKK6* via a negative-feedback loop mechanism. (This figure is available in colour at *JXB* online.)

In plants, feedback loops are important regulatory mechanisms during development and stress responses. In Arabidopsis, the bHLH transcription factor *HBI1* functions as a major node in a complex feedback loop that mediates the trade-off between growth and immunity (Fan *et al.*, 2014). Another bHLH transcript factor, *MYC2*, is a component of the MKK3–MPK6–MYC2 feedback loop, which is involved in blue-light-mediated seedling development (Sethi *et al.*, 2014). As with many transcription factors, miRNAs also play important roles in feedback loop mechanisms. Arabidopsis miR171 and its target scarecrow-like proteins constitute a feedback loop that is critical for mediating gibberellin–DELLA signalling in light (Ma *et al.*, 2014). In the current study, we report a new feedback loop that includes miR5272a and the MAPK cascade (Fig. 7). When *F. oxysporum* infects cotton, *GhMKK6*-mediated signal transduction is activated. This signalling pathway induces the expression of downstream resistance genes, ROS production, and SA- and/or JA-mediated defence responses (see Supplementary Fig. S12). Similar to SA-mediated defence responses, pathogen-activated respiratory bursts have also been implicated in controlling HR (Yun *et al.*, 2011). Uncontrolled HR harms plants, and in cotton ghr-miR5272a expression acts to prevent an excessive HR by regulating *GhMKK6* expression after *F. oxysporum* infection. Taken together, our data suggest that *GhMKK6* is critical for cotton disease resistance and that the regulatory function of ghr-miR5272a in the immune response is based on its *GhMKK6* mRNA-cleavage activity.

## Supplementary data

Supplementary data are available at *JXB* online.

Fig. S1. Relative expression of *GhMKK6* in different tissues.

Fig. S2. Expression levels of SA- and JA-mediated defence pathway genes in *GhMKK6*-silenced cotton after *F. oxysporum* infection.

Fig. S3. Silencing of *GhMKK6* by the fragment from nucleotides 136 to 522 in cotton reduces resistance to *F. oxysporum*.

Fig. S4. Expression levels of *GhMKK6* in transgenic lines.

Fig. S5. The activation of MAPKs in empty vector and transgenic tobacco lines with or without *F. oxysporum* infection.

Fig. S6. Expression levels of SA- and JA-mediated defence pathway genes in *GhMKK6*-overexpressing tobacco 4 h after *F. oxysporum* infection.

Fig. S7. The activity of GhMKK6EE and GhMKK6AA.

Fig. S8. Expression levels of GhMKK6 in different co-transformed tobacco leaves.

Fig. S9. Prediction of miR5272a targets in tobacco.

Fig. S10. The expression pattern of ghr-miR5272a in cotton under SA treatment.

Fig. S11. Overexpression of a ghr-miR5272a mimic with imperfect binding sites inhibits the function of ghr-miR5272a.

Fig. S12. The expression levels of SA- and JA-mediated defence pathway genes in wild-type cotton.

Table S1. Oligonucleotide primers used in gene cloning, vector construction, and qPCR.

Table S2. Oligonucleotide primers used in qRT-PCR.

## Supplementary Material

supplementary_figures_S1_S12

supplementary_tables_S1_S2
