# Supplementary material for: ghr-miR5272a-mediated regulation of GhMKK6 gene transcription contributes to the immune response in cotton
Source: J Exp Bot. 2017 Oct 21;68(21-22):5895–906. doi: 10.1093/jxb/erx373 (PMC5854127; doi:10.1093/jxb/erx373)
Supplement: supplementary_figures_S1_S12 [file erx373_suppl_supplementary_figures_s1_s12.pdf]

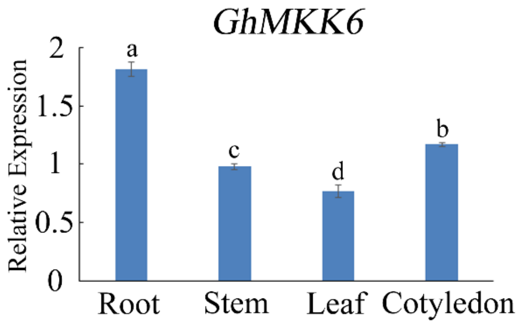

Fig. S1. The expression pattern of *GhMKK6* in different tissues. The data are the mean ± standard error (SE) of three independent experiments. The letters above the columns represent significant differences ($P < 0.05$) based on Tukey HSD test.

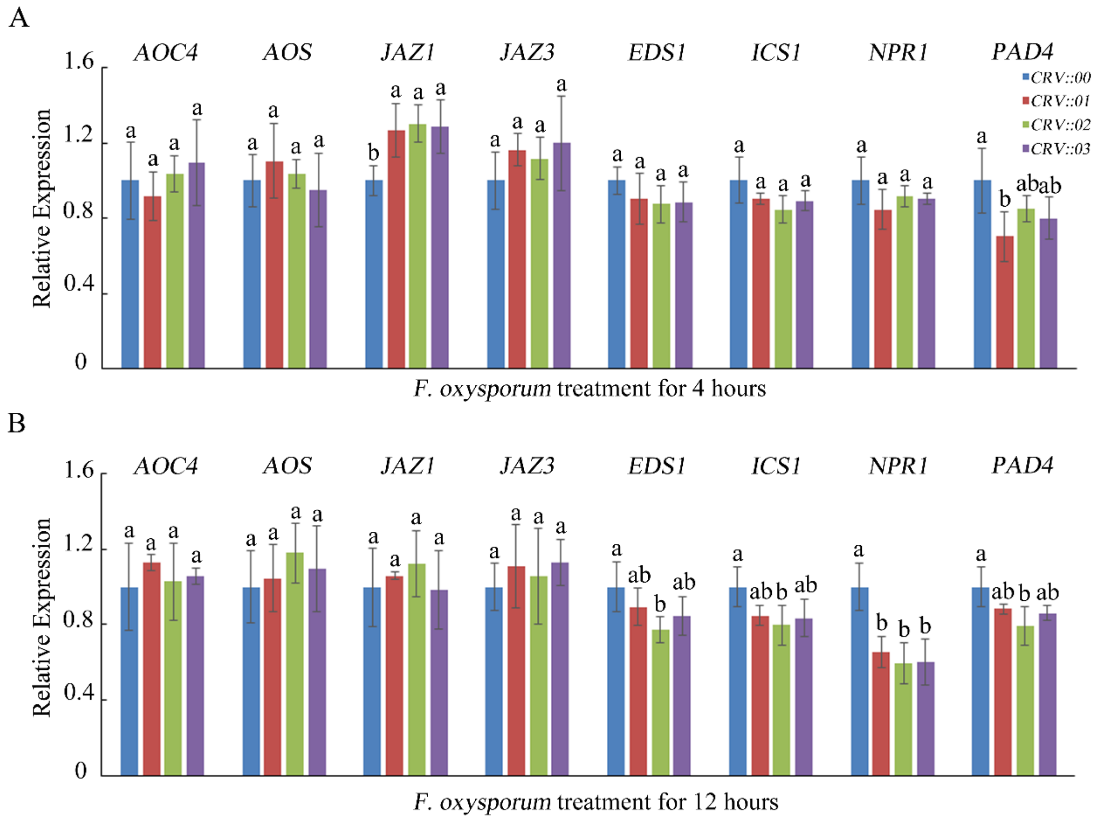

Fig. S2. The expression levels of SA- and JA-mediated defence pathway genes in *GhMKK6*-silenced cotton after *F. oxysporum* infection. A and B. qRT-PCR analysis for SA- and/or JA-mediated defence pathway gene expression in *CRV::00*, *CRV::01*, *CRV::02* and *CRV::03* after *F. oxysporum* infection for four hours or twelve hours. The Error bars indicate the mean values ± SE of three independent experiments (n=6). The letters above the columns represent significant differences ($P < 0.05$) based on Tukey HSD test.

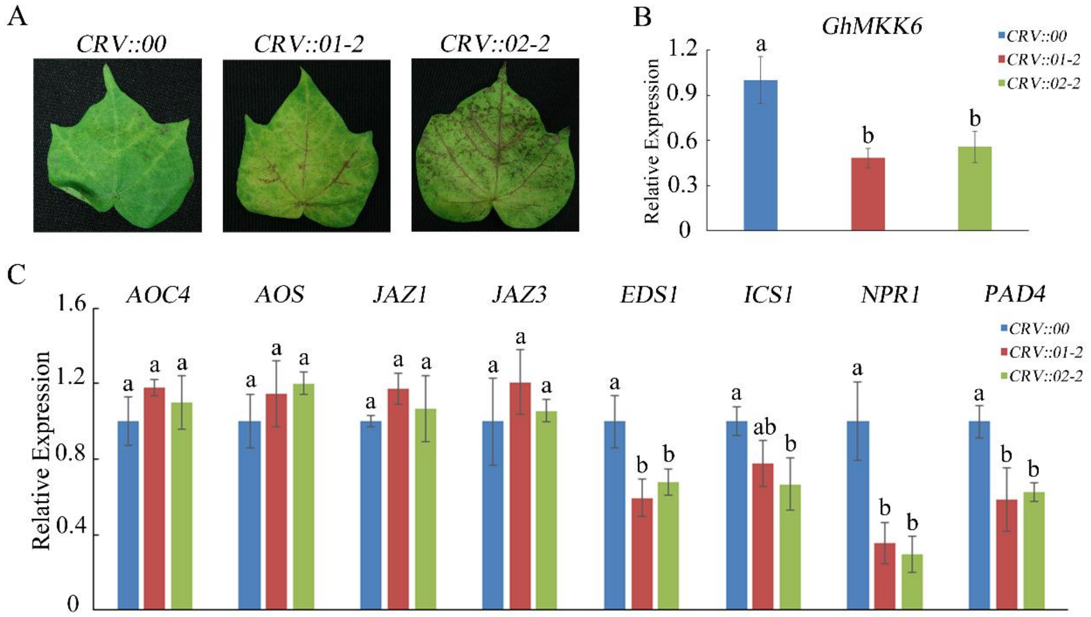

Fig. S3. Silencing of *GhMKK6* by the fragment from nucleotides 136 to 522 in cotton reduces resistance to *F. oxysporum*. A. Representative phenotypes of *GhMKK6*-silenced cotton infected with *F. oxysporum* for five days. **B.** *GhMKK6* RNA levels in *GhMKK6*-silenced cotton. **C.** qRT-PCR analysis for SA- and/or JA-mediated defence pathway gene expression in *CRV::00*, *CRV::01-2* and *CRV::02-2* after *F. oxysporum* infection for five days. *CRV::00* was the empty vector control. The Error bars in (B) and (C) indicate the mean values ± SE of three independent experiments (n=6). The letters above the columns represent significant differences ($P < 0.05$) based on Tukey HSD test.

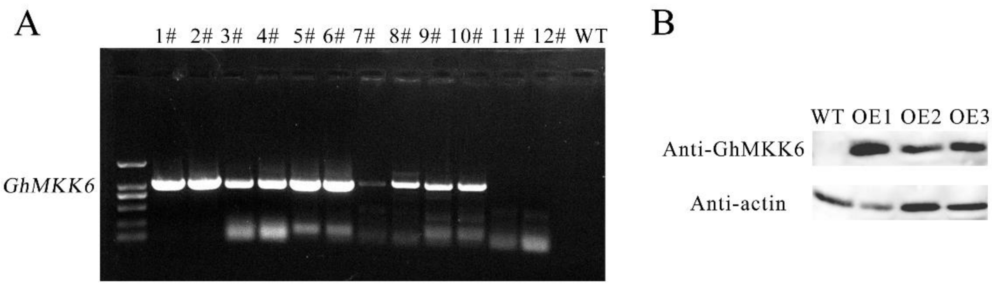

Fig. S4. The expression level of *GhMKK6* in transgenic lines. A. The ORF of *GhMKK6* was insect into the genome of tobacco. **B.** The protein level of GhMKK6 in OE1, OE2 and OE3 transgenic lines obtained from T_3 progeny.

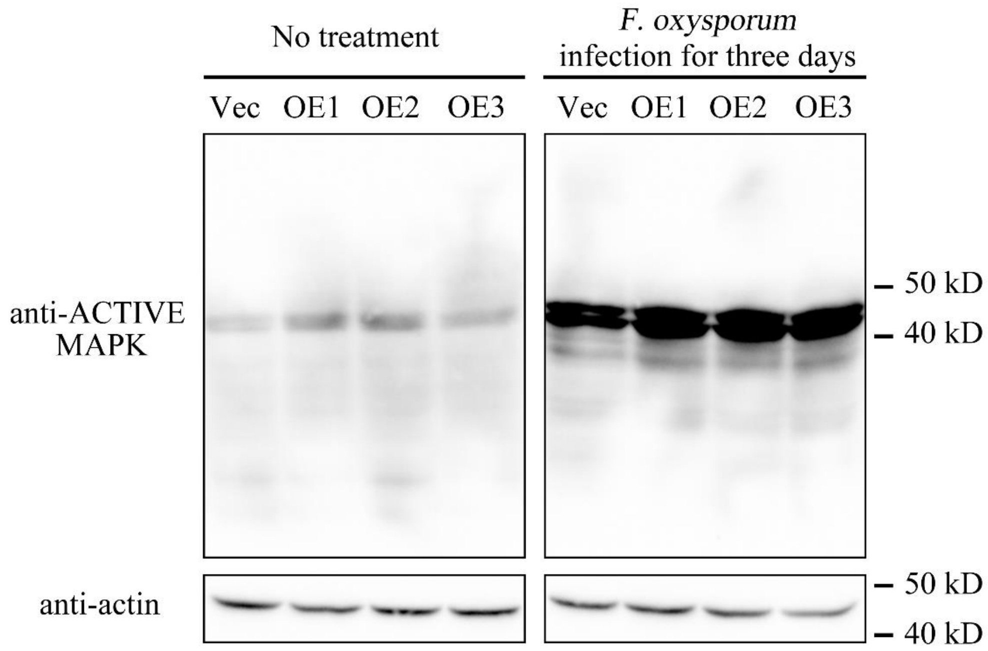

Fig. S5. The activation of MAPKs in Vec and transgenic tobaccos with or without *F. oxysporum* infection. Few MAPKs were activated in Vec or transgenic plants without *F. oxysporum* infection. After *F. oxysporum* infection for three days, the MAPKs were dramatically activated. And the phosphorylation level of MAPKs in transgenic plants show much higher than those in Vec plants.

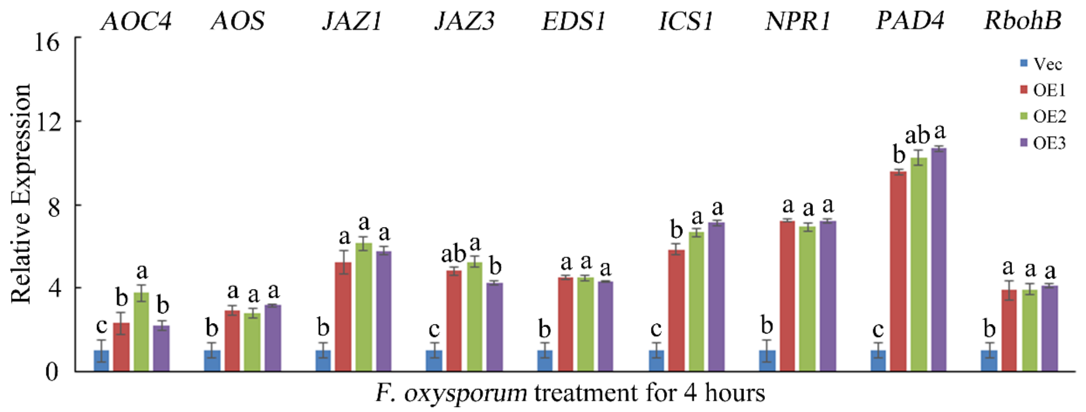

Fig. S6. The expression levels of SA- and JA-mediated defence pathway genes in *GhMKK6*-overexpressing tobacco after *F. oxysporum* infection for four hours. The Error bars indicate the mean values ± SE of three independent experiments (n=6). The letters above the columns represent significant differences ($P < 0.05$) based on Tukey HSD test.

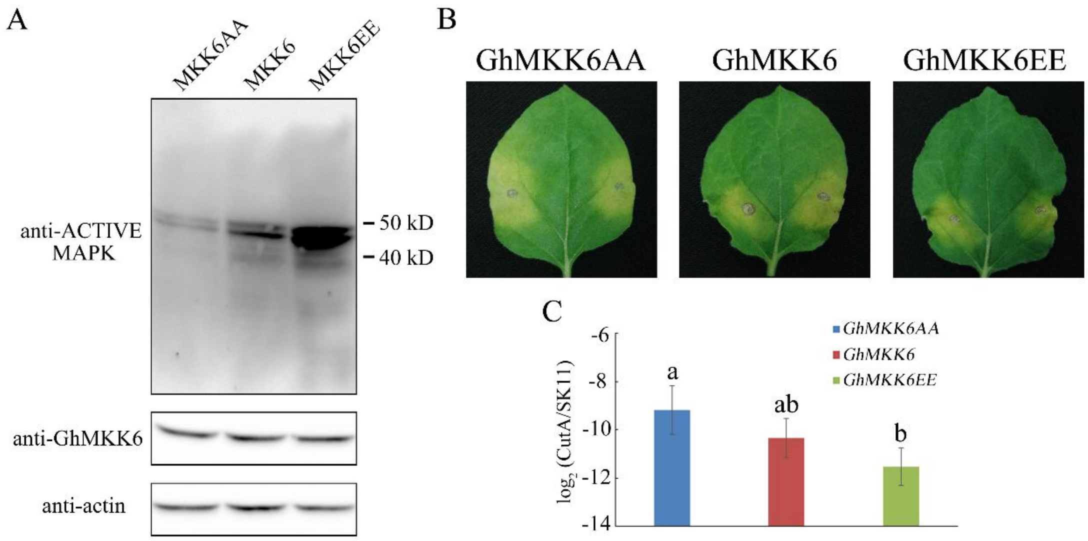

Fig. S7. The activity of GhMKK6EE and GhMKK6AA. A. The protein level of activated MAPKs in leaves expressing *GhMKK6EE*, *GhMKK6* or *GhMKK6AA*. Western blot was used to test the activity of MKK6EE and MKK6AA via the Anti-pTEpY phospho-p44/42 MAPK. **B.** Representative phenotypes of plants infect *F. oxysporum* for three days after transiently expressing *GhMKK6*, *GhMKK6AA* or *GhMKK6EE*. **C.** Pathogen disease index in plants after *F. oxysporum* infection for three days. The Error bars indicate the mean values ± SE of three independent experiments (n=6). The letters above the columns represent significant differences ($P < 0.05$) based on Tukey HSD test.

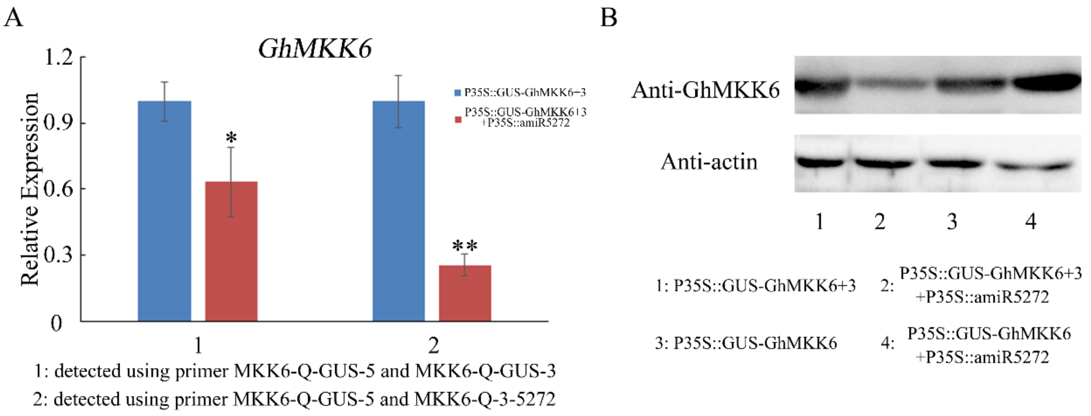

Fig. S8. The expression level of GhMKK6 in different co-transformed tobacco leaves. A. The expression level of *GhMKK6* in tobacco leaves transformed with 35S::GUS-GhMKK6+3 or co-transformed with 35S::GUS-GhMKK6+3 and 35S::amiR5272. Section 1, qPCR was performed using the primer MKK6-Q-GUS-5 (from nucleotides 915 to 934) and MKK6-Q-GUS-3 (the reverse complementary sequence of nucleotides 1232 to 1253); section 2, qPCR was performed using the primer MKK6-Q-GUS-5 and MKK6-Q-3-5272 (the reverse complementary sequence of the ghr-miR5272a binding site). The Error bars indicate the mean values ± SE of three independent experiments (n=6). Asterisks (* or **) above lines indicate significant differences (*P < 0.05; **P < 0.01) based on Tukey HSD test. **B.** The protein level of GhMKK6 in different co-transformed tobacco leaves.

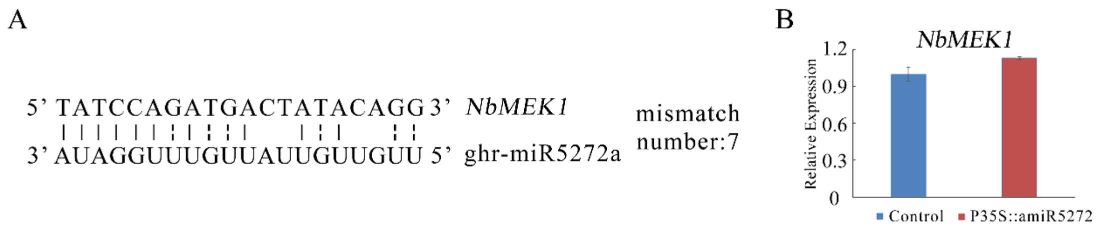

Fig. S9. Prediction of miR5272a targets in tobacco. A. Predication of miR5272a target in tobacco. **B.** The expression level of *NbMEK1* in tobacco after amiR5272 transformation. The Error bars indicate the mean values ± SE of three independent experiments (n=6). Asterisks (* or **) above lines indicate significant differences (*P < 0.05; **P < 0.01) based on Tukey HSD test.

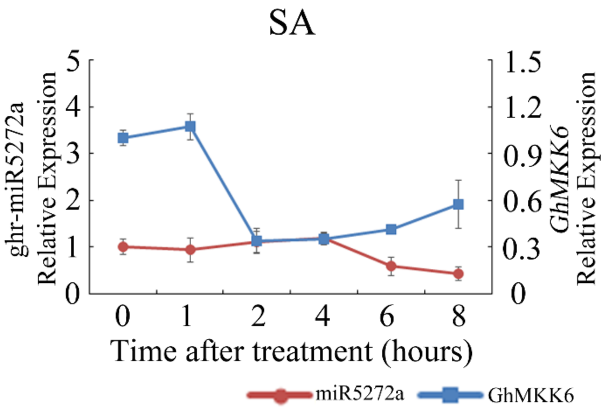

Fig. S10. The expression pattern of ghr-miR5272a in cotton under SA treatments. Cotton cotyledons were sprayed with SA (10 mM) and collected from cotton plants, frozen in liquid nitrogen, and stored at -80°C for RNA extraction. The Error bars indicate the mean values ± SE of three independent experiments (n=6).

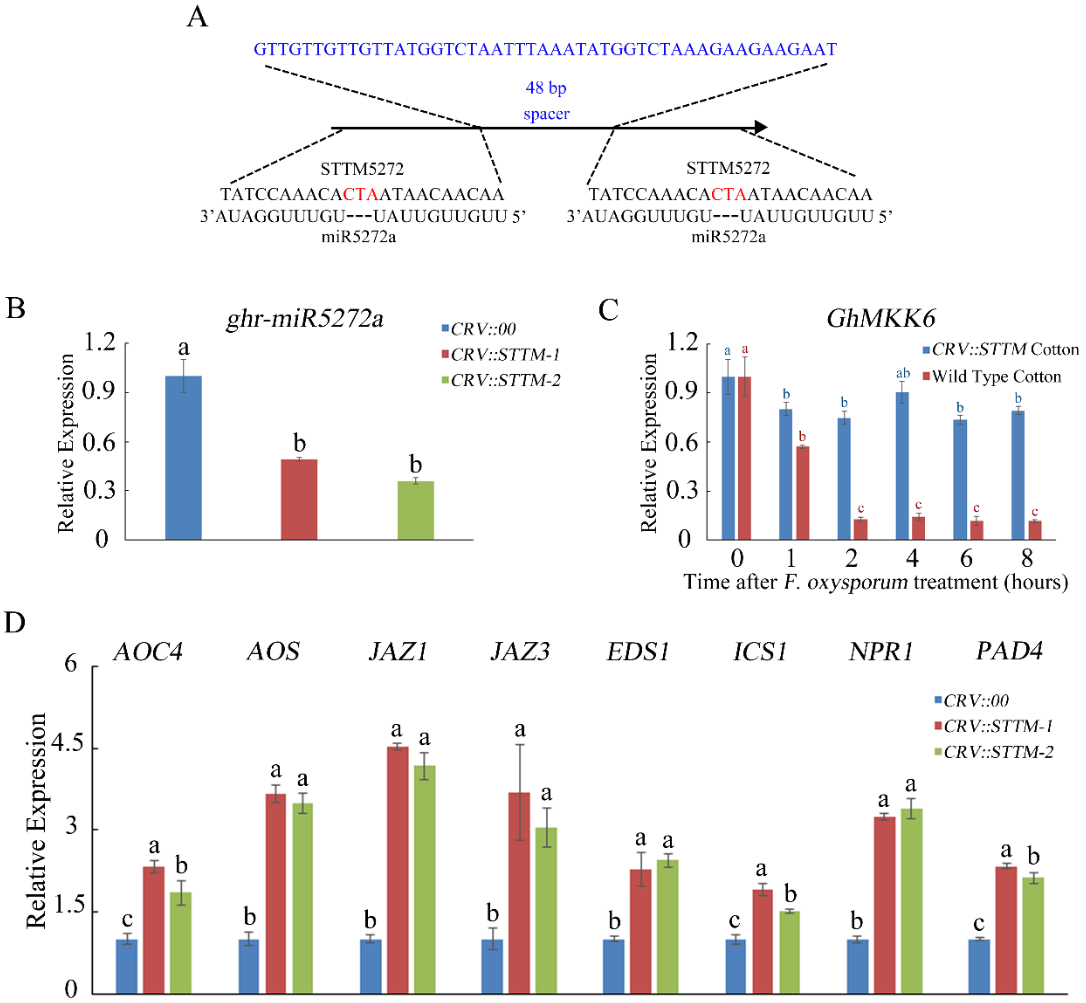

Fig. S11. Overexpression of ghr-miR5272a mimic inhibits the function of ghr-miR5272a. A. Sequence of the STTM inserted into pCLCrVA-STTM. This STTM contains two imperfect ghr-miR5272a binding sites, separated by a 48-bp spacer. **B.** The expression level of ghr-miR5272a in pCLCrVA-STTM cotton. **C.** The expression level of *GhMKK6* in pCLCrVA-STTM cotton after *F. oxysporum* infection. The Error bars indicate the mean values ± SE of three independent experiments (n=9). The letters above the columns represent significant differences ($P < 0.01$) based on Tukey HSD test. **D.** qRT-PCR analysis of SA- and JA-mediated defence pathway gene expression in pCLCrVA-STTM cotton after *F. oxysporum* infection for 5 days. The Error bars in (B) and (D) indicate the mean values ± SE of three independent experiments (n=9). The letters above the columns represent significant differences ($P < 0.05$) based on Tukey HSD test.

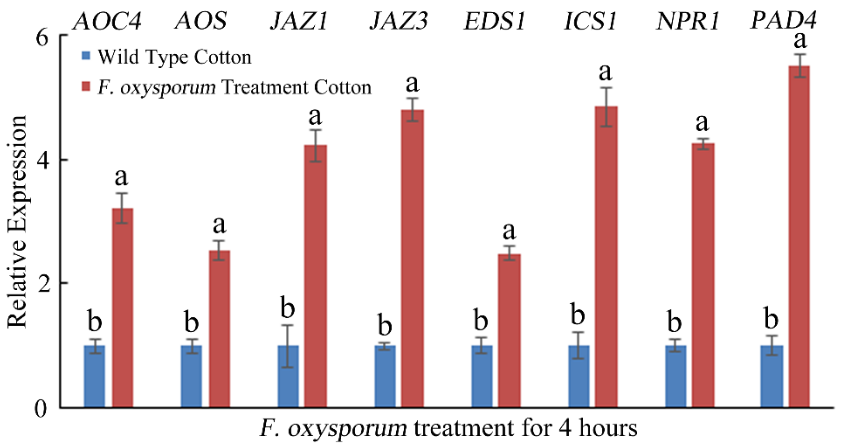

Fig. S12. The expression levels of SA- or JA-mediated defence pathway genes in wild-type cotton. For *F. oxysporum* treatment, cotton seedlings were inoculated with conidial *F. oxysporum* suspensions (10^6 conidia/mL) using the root-dip method. The Error bars indicate the mean values ± SE of three independent experiments (n=3). The letters above the columns represent significant differences ($P < 0.05$) based on Tukey HSD test.
