# Supplementary material for: ghr-miR5272a-mediated regulation of GhMKK6 gene transcription contributes to the immune response in cotton
Source: J Exp Bot. 2017 Oct 21;68(21-22):5895–906. doi: 10.1093/jxb/erx373 (PMC5854127; doi:10.1093/jxb/erx373)
Supplement: supplementary_tables_S1_S2 [file erx373_suppl_supplementary_tables_s1_s2.pdf]

Tables S1. Oligonucleotide primers used in gene cloning, vectors constructing and qPCR.

Primer	Primer sequence (5'→3')
The full-length cDNA primers	
MEK6-QC-5	GAACCCTAACGGAACAGTTATAAAA
MEK6-QC-3	TTGTTATATTTGTATGGATACATATTTA
Vector construct primers	
MEK6-PRI-5	CATATGAAGAGCAAGAAGCCATTGAAGC
MEK6-PRI-3	GGTACCTTATCTTGGGTAATTCACAGG
Primers used in qRT-PCR	
MEK6-Q-5	CCTATCTCTTCTTTCTTGACGGCG
MEK6-Q-3	CTTCAAGGCAAACAATCTTCCAACC
Ghr-miR5272a	TTGTTGTTATTGTTTGGATA
U6	GATGACACGCACAAATCGAGAAAT
UBI-F	CCAGAAGGAATCCACTTTGC
UBI-R	CCAGCTCACATCAGCATACG
NtActin-5	TGGACTCTGGTGATGGTGTC
NtActin-3	CCTCCAATCCAAACACTGTA
MEK6-Q-GUS-5	CCTCCACCAACTGCTCCATC
MEK6-Q-GUS-3	CATATTTATATCCGACACTCAC
MEK6-Q-3-5272	TTGTTATATTTGTATGGATA
FoCuT-Q-5	GACTGACTTGATAGGGAACGC
FoCuT-Q-3	CACGGTATGCTCCACCAACCTAG
Site-directed mutagenesis primers	
MEK6-S219E-5	CAATGCTAGCTAGCGAAATGGGCCAGAGAG
MEK6-S219E-3	CACTGACACCAAAATCAGTGATCTTC
MEK6-T225E-5	TGGGCCAGAGAGATGAATTTGTTGGG
MEK6-T225E-3	TTTCGCTAGCTAGCATTGCACTGAC
MEK6-S219A-5	CAATGCTAGCTAGCGCTATGGGCCAGAGAG
MEK6-T225A-5	TGGGCCAGAGAGATGCATTTGTTGGG
MEK6-T225A-3	TAGCGCTAGCTAGCATTGCACTGAC
pCLCrV vector construct primers	
MEK6-CRV-5	ACTAGTCTATCACAATGGGGCCATATCTCTGG
MEK6-CRV-3	TTAATTAAGATGGAGCAGTTGGTGGAGGC
MEK6-CRV-52	ACTAGTGGACATTTCATGATGGCG
MEK6-CRV-32	TTAATTAAGTCTGATCACATCGGCCAGAG
amiR5272-CRV-5	ACTAGTCATGGTTTTTCGCTTGCTAGG
amiR5272-CRV-3	TTAATTAACCATGGCGATGCCTTAAATAAAG
Primers used in GUS assay	
PRI-GUS-5	CATATGTTACGTCCTGTAGAAACCC
PRI-GUS-3	GAGCTCTTGTTTGCCTCCCTGCTGC
MEK6-GUS+3-3	GGTACCTTGTTATATTTGTATGGATACATATTTATATC
MiR5272-GUS-5	CATATGCATGGTTTTTCGCTTGCTAGGTTATAG
MiR5272-GUS-3	GAGCTCCCATGGCGATGCCTTAAATAAAGAT

Tables S2. Oligonucleotide primers used in qRT-PCR

Primer	Primer sequence (5'→3')
GhNPR1-5	GCGAATCGTTGCTTTCTTCTTCA
GhNPR1-3	CACGTGGTGCTGTTGTTGTTACTG
GhICS1-5	ATGGATGAATGGGTGCGAAGG
GhICS1-3	AAGAATGCCAGAGGTAAGAGGAGGA
GhEDS1-5	GGCAGACCAAGACGCTACAGATACA
GhEDS1-3	GCAGCAACAGCTCCTCTACCTCAA
GhPAD4-5	GGATGGAAGAATGGAAAGAAATGAA
GhPAD4-3	GAACTAGGAAAGCAGACTAAGGAACCA
GhJAZ1-5	GACCAAATCTTGTGGCATCTACCTC
GhJAZ1-3	CTTTGGTTCCACTGCTGCTGATT
GhJAZ3-5	GCGGGTGAAGTGAATGTCTTTGAT
GhJAZ3-3	GAACTGGTTTCAAAATAGGAGTCTGGA
GhAOS-5	ATCCGACGGTGGAGAATAAACAGT
GhAOS-3	CGATGTCCCAACTTCAATCTCAAAC
GhAOC4-5	AATAGAGCATAAACCCGAAATGAAAG
GhAOC4-3	CAAAAATGCCAGACCCACCAGTA
GhrbohB-5	GAAGAACTTGATCCAGATAATGCCGG
GhrbohB-3	CGGTACTGCACGAACTTATACGCG
GhSK11-5	CTGACTGTGTGGTTAGTGCAATGC
GhSK11-3	CTCCTTTCATTGAGACATTCCAC
NtMEK1-5	CCATCAAAGTCATCGGGAAGGG
NtMEK1-3	CCCACGGTCCATATACTCAAGAACC
NtNPR1-5	GCAGCAGACGATGTAATGATGG
NtNPR1-3	TCCACAAGCCTAGTGAGCCTC
NtICS1-5	CAATTCCGCCATCTCTCACT
NtICS1-3	TGAGCATGAAGCCACTCAAG
NtEDS1-5	TCTGGATAGGCTGAAAGCAC
NtEDS1-3	CCATACAAGCAAAGCAGTTCC
NtPAD4-5	GGACTCACACTCCAGCGTTT
NtPAD4-3	GGCAACTCATCCTCTTCCTG
NtJAZ1-5	GTCACCGGCCAGAAGTCTC
NtJAZ1-3	TGGCACCTGAGTTCGCGTAC
NtJAZ3-5	CTGAGGCAAAATCTGAACCGGAG
NtJAZ3-3	GCACCCAATCCAAGCCACAC
NtAOS-5	CTTCTCTTCGCTACGTGTTTCAATTCC
NtAOS-3	CTCCATCGCCAACATCGTGATC
NtAOC4-5	GGAATAACGGCTGGACTCTGC
NtAOC4-3	CTGAACGGCGATGTGACCATAATC
NtrbohB-5	GTTTGCCAGCCACCACCTAAT
NtrbohB-3	AAGAGCAGAACGAGCATCACC
NtSK11-5	CTTCCCTGCGATTCTCTCTCTC
NtSK11-3	CTCAAACCGGATACAGGTGCTAAG
